# Development of a core outcome set for trials for management of oral submucous fibrosis (OSFCOS): A consensus study protocol

**DOI:** 10.1371/journal.pone.0325158

**Published:** 2025-07-30

**Authors:** Shailesh M. Gondivkar, Monal Yuwanati, Sachin C. Sarode, Amol R. Gadbail, Vidya Lohe

**Affiliations:** 1 Department of Oral Medicine & Radiology, Government Dental College & Hospital, Nagpur, Maharashtra, India; 2 Department of Oral Pathology & Microbiology, Saveetha Dental College and Hospital, Saveetha Institute of Medical and Technical Sciences, Saveetha University, Chennai, Tamil Nadu, India; 3 Department of Oral Pathology & Microbiology, Dr. D.Y. Patil Dental College & Hospital, Dr. D. Y. Patil Vidyapeeth, Pune, Maharashtra State, India; 4 Department of Dentistry, Government Medical College & Hospital, Nagpur, Maharashtra State, India; 5 Department of Oral Medicine & Radiology, Sharad Pawar Dental College & Hospital, DMIHER (Deemed to be University), Sawangi (M), Wardha, Maharashtra State, India; University of the Republic Uruguay: Universidad de la Republica Uruguay, URUGUAY

## Abstract

**Background:**

The effectiveness of oral submucous fibrosis (OSF) treatment is currently assessed with a broad range of clinical outcome measures. This heterogeneity complicates study comparison, synthesis of results, and evidence-based clinical practice guideline development. A core outcome set (COS) is a set of agreed, standardized outcomes that will be measured and reported across all clinical trials for a particular condition.

**Objective:**

Our aim is to create a COS (OSFCOS) for efficacy trials that look at the management OSF.

**Methods:**

An initial list of the potentially relevant outcomes will be drawn by a systematic review of randomized controlled trials focused on OSF treatment. An e-Delphi process will be done to obtain agreement among important stakeholders. The stakeholders will consist of OSF patients, members of the Indian Academy of Oral Medicine & Radiology, the representatives from the Indian Dental Association, clinical researchers, and other oral health specialists. Participants will be asked to rate the importance of each outcome on a structured online questionnaire. Participants will also be allowed to propose new outcomes in Round 1. Feedback and the aggregated scores will be given in later rounds to guide re-rating. If after the second round, there is still no consensus, a third round will be taken.Final consensus on outcome inclusion will be determined based on predefined criteria.

**Conclusion:**

This protocol outlines a structured, inclusive approach to developing a core outcome set for OSF. The finalized OSFCOS will be made publicly available to guide outcome selection in clinical trials, improve the quality of systematic reviews, and support evidence-based clinical recommendations for the treatment of OSF.

## Introduction

Oral submucous fibrosis (OSF) is a chronic, persistent condition linked to chewing betel nut that is clinically characterized by oral mucosa fibrosis and restricted mouth opening. Due to the immigrant population, it is not just restricted to Asian countries but is also expanding throughout the world and has turned into a global health issue [1,2]. OSF symptoms include limited mouth opening, burning sensation, palpable fibrous bands, loss of mucosal flexibility, mucosal blanching, constrained tongue movements, and shrunken or bent uvula [3,4]. Along with this, different degrees of severity of hearing loss, speech difficulties, and taste sensation loss have also been recorded in the literature [5]. OSF has significant detrimental consequences on quality of life, including functional limitations. Psycho-social issues, such as anxiety, low self-esteem, and social isolation, are also affected. Evidence from the literature indicates that the combination of these burdens becomes increasingly worse with disease progression, resulting in a diminished quality of life [6,7]. The classification of OSF as an oral potentially malignant disorder (OPMD) by the WHO Collaborating Center for Oral Cancer [8] has been confirmed in their most recent report [9]. A recent umbrella review on OSF reveals a malignant transformation rate of 4.2% to 6% [10].

Despite the fact that numerous treatment strategies have been explored in the past, none have been shown to be a superior choice for the holistic management of OSF symptoms [11]. There is a dearth of high-quality evidence addressing the management of OSF, according to previous systematic reviews [11,12]. As a result, there is currently a great deal of debate surrounding the standardization of OSF therapy, and it is unclear which OSF management is best and under what conditions, which results in the wasteful use of healthcare resources and subpar results. This uncertainty is exacerbated by the large number of outcomes utilized in clinical trials, which makes it difficult to compare or combine the findings of many studies [13]. Even in high-quality trials, the use of such varied outcome measures or measuring techniques can compromise the comparison of these trials’ findings and prevent the therapeutic advantages of a specific intervention from being revealed with any certainty [11]. Furthermore, such variability may hinder the synthesis of the available evidence and prohibit the pooling of study data for a thorough analysis or meta-analysis. As a result, in the current era of evidence-based clinical practice, new knowledge is not successfully adopted by the scientific community, and the best possible care is not provided to patients. There is currently no agreement on the outcomes that ought to be evaluated when examining therapies for OSF.

The COMET (Core Outcome Measures in Effectiveness Trials) initiative (http://www.comet-initiative.org) brings together researchers interested in the creation, application, and promotion of core outcome sets (COS), which are defined as an agreed-upon, standardized collection of outcomes that should be measured and reported in all trials for a particular clinical area. The COS serves as the minimum that should be assessed and reported in every trial and are also appropriate for other kinds of audit and study. Complete and on-going COS protocols and papers are kept in a COMET database that is freely available to the public.

In addition to supporting future data synthesis and sharing, the creation of a consensus-selected set of outcomes for OSF will encourage clinical researchers and trialists to choose outcomes that are relevant to patients and clinicians.Creation of a COS, thus, will help facilitate improved outcome reporting for OSF trials and will assist in the comparison of competing research projects. It will support the integration of knowledge and will guide decisions pertaining to clinical measures as well as formulate therapeutic recommendations. This may, in the end, minimize redundant research and optimize patient treatment care quality.

The aim of this study will be to develop a core outcome set (OSFCOS) for trials for management of OSF. The following are the objectives of the current study: (i) To find possibly pertinent outcomes for OSF in the currentliterature and by interviewing key stakeholders; (ii)To achieve consensus across specialists in the field, patients and public on a COS for management of OSF by using the Delphi survey technique and consensus meeting.

The COS will be suitable to research trials examining the management of OSF in primary or secondary care. It won’t be constrained by age, physical condition, or geographical location of trials. The COS will not be appropriate to the treatment of OSF patients who also have concomitant oral mucosal lesions or OSF associated oral squamous cell carcinoma.

## Materials and methods

**Fig 1:**
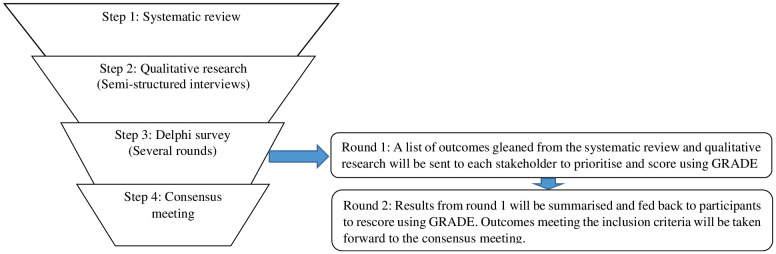
Multi-step process used to develop the COS for trials for management of OSF.

The study has been registered on the COMET database (Registration no. 2804; http://www.comet-initiative.org/studies/details/2804?result=true). The development of the present study protocol will be guided by the COMET handbook [14] and is reported in accordancewith the Core Outcomes Set-STAndardised Protocol Items (COS-STAP) (Table S1).A multi-step process (Fig 1) using consensus procedure with key stakeholders will be used to develop a COS for OSF management.

### Ethics Statement

The Institutional Ethics Committee of Government Dental College & Hospital, Nagpur, India provided ethical approval for the study (reference IECGDCHN/14/24). Participants will be requested to submit written informed consent.

### Step 1- Identification of potentially relevant outcomes from existing literature

As a part of this study, a systematic review of randomized controlled trials on the treatment of OSF was conducted in order to compile a preliminary list of possibly pertinent outcomes. Previous publication have provided a detailed description of the databases searched, inclusion and/or exclusion criteria, data extraction, and trials obtained [13]. The list of outcome domains and outcome measures (both patient reported and clinician observed) reported in the systematic review is presented in the Table 1.

**Table 1:** List of outcome domains and outcomes identified in the systematic review.

Outcome domains	Outcome measures
**Clinical symptoms**	Mouth opening
	Burning sensation
	Tongue protrusion
	Cheek flexibility
	Oral symptoms
	Recurrent ulceration
	Symptom score
	Pain upon mouth opening
	Shrunken/deviated uvula
	Difficulties in speech
	Difficulties in swallowing
	Tinnitus
	Suppleness and elasticity of the buccal mucosa
**Clinical scores**	Sign score
	Clinical staging
	Severity rating
	Histopathological score
**Clinical response**	Treatment response
**Adverse effect**	Side effect
	Adverse effect/reaction

### Step 2- Identification of additional outcomes: involvement from key stakeholders

Stakeholders are key to identifying, ranking, and agreeing on key outcomes using interviews, focus groups, Delphi surveys, and a consensus meeting at the end. Their participation guarantee that the core outcome set is relevant, patient-focused and broadly applicable, making it more acceptable and implementable in the future in clinical research and practice.

The outcomes that matter to various stakeholder groups can be determined using qualitative data collection techniques [15]. Therefore, semi-structured interviews with key stakeholders will be conducted in order to ascertain the outcomes they value most and to rank the outcomes produced by the systematic review. Focus groups will allow for participant interaction and idea sharing while fostering group consensus on critical OSF issues. Through interviews, patients will be able to express their experiences in their own words and according to their own framework. Focus groups and interviews are expected to yield a depth of data that is not anticipated from more straightforward questionnaire-based surveys. The qualitative components of the research will be conducted using an iterative methodology.

#### Inclusion criteria.

Patients with a clinical diagnosis of OSF who are at least 18 years old, receiving therapy, and willing to take part in the study will be enrolled.

#### Exclusion criteria.

Patients with any systemic disorders, oral lesions other than OSF, and those with malignant alterations present will beexcluded.

#### Sampling and recruitment.

The research subjects will be selected specifically from the outdoor patient department of the Oral Medicine and Radiology at the Government Dental College and Hospital in Nagpur, India. Purposive sampling will be used to identify participants with various levels of OSF severity and a variety of perspectives on the results of OSF treatment. There will be enough participants enrolled to reach information saturation, but not enough to prevent in-depth analysis.Based on earlier studies of a similar kind [16,17], a total sample size of around 25–30 participants will be obtained through the use of three to four focus groups (each with five to six participants) and interviews with about twelve OSF patients. However, if fresh viewpoints keep surfacing, the precise qualitative sample size might grow.

#### Recruitment.

A verbal description of the study will be given to prospective volunteers when they are approached. Separate written information sheets will also be provided to attendees to peruse afterwards. On the interview day, written consent will be acquired. The freedom to leave the study at any moment will be communicated to participants.

#### Data collection.

At a mutually convenient time and location in a non-clinical setting, focus groups and interviews will be scheduled. In order to protect patient privacy at all times and promote candor among interview subjects, all interviews will be done using pseudonyms. Participants are invited to explore their oral health concerns in detail during focus group sessions. Following the completion of consent forms, participants’ postcodes will be used to collect socioeconomic demographic data.

#### Topic guide.

The topic guide will be piloted first, then revised as necessary. The proposed topic guides for semi-structured interviews and focus group discussions are reported in the Tables 2 and 3 respectively. The interviewer will be flexible in changing the interview topics and open to the participants’ narratives. The interviews and conversations will last for 20–30 minutes and be recorded. Breaks are welcome at any time for participants. An expert qualitative researcher will serve as a co-facilitator for each focus group and assist in conducting them.

**Table 2:** Semi-Structured interview guide.

**Introduction**	Hello, How are you today? Thank the participant for their time
	Explain the purpose of the study.
	Inform the participant that participation is voluntary and Confidentiality of the interviews
	Get informed consent
**Background Information**	Would you mind sharing a little about your experience with OSF?
	How long has it been since you started experiencing symptoms of OSF?
	Have you undergone any form of treatment or intervention for OSF?
	In what ways has OSF impacted your daily life and overall wellbeing?
**Core Outcomes in OSF Management**	What are the most essential gains you want to observe from treatment?
	What problems or symptoms are of greatest concern to you?
	What specific areas of your wellbeing or health are relevant to you that should be evaluated in clinical trials?
	Are there any symptoms that you think are often ignored in terms of study or treatment but you possibly notice?
	What aspects would make a significant difference in your normal daily activities?
**Difficulties in Choosing and Measuring Outcomes**	Do healthcare professionals’ assessments of your condition differ from one another?
	Are the main issues that bother you being addressed with the existing treatment?
	Is there any established method to evaluate how useful a treatment is?
**Additional Insights**	In your opinion, should OSF research address any more findings or issues?
	What other comments do you have concerning your OSF experience?
**Closing**	Express gratitude to the participant for sharing their thoughts and time

**Table 3:** Focus group discussion guide.

**Introduction**	Greet the participants and introductions of the facilitator
	Describe the goal of the study
	State that participation is voluntary and Confidentiality of the interviews
	Get informed consent
	Encourage participants to be open and honest
**Background Information**	Could everyone briefly describe their experience with OSF?
	What impact has OSF had on your day-to-day life?
**Identifying Core Outcomes in OSF**	Which symptoms are the most troublesome to you?
	How everyday activities do are impacted by OSF? (Encourage discussion on eating, talking, brushing teeth and interacting with others)
	Why do you consider these outcomes significant? (Encourage discussion on psychological, emotional, and social aspects)
	Which of the following outcomes do you consider most important? (Enlist outcomes)
	What therapies have you had for OSF?
	In what ways did the treatment meet your expectations?
	Did you find any difficulties or adverse effects to be concerning?
	Do you believe any other outcomes that should be measured that are not being addressed?
**Ranking and Prioritization of Outcomes**	List the important results discovered throughout our talk
	Can we rate these items from most to least important?
	Why do you believe the top-ranked outcomes are critical?
**Final Thoughts**	Summarize key points discussed.
	Would you want to add anything further about your experiences?
**Closing**	Express gratitude to the participant for sharing their thoughts and time

#### Data analysis.

Focus group and interview field notes will be recorded and verbatim typed into a transcript. A thematic analysis will be performedas recommended by Braun and Clarke [18]. This will entail creating a list of essential codes, which will result in the creation of a coding scheme. All transcripts will use this coding system. After that, codes will be categorized, leading to the development of important topics. The results of each of the themes will then be determined. Data analysis will be done by the same researcher who performed the interviews. A 10% verification check will be performed on a subset of transcripts by a second researcher.

A long list of outcomes will be created from the findings of the systematic review and the qualitative interviews. After debate within the research team, each result will be assigned to the relevant domain. A proper outcome domain framework will be taken into consideration in order to speed up the process. The medical jargon will be enclosed in brackets and all results will be expressed in lay language. There will also be a succinct explanation of the result.

### Step3- Delphi surveys

We aim to include stakeholders across three different stakeholder groups: 1) OSF patients; 2) clinicians; and 3) researchers/experts. For OSF patients, the pertinent clinicians, and researchers/experts, three 3-round Delphi surveys will be conducted. The lists of outcomes generated in stages 1 and 2 will be cross-referenced and expanded into a lengthy list of items so that participants may assess the relevance of the results on a 9-point scale. The study team will discuss the findings and make any necessary revisions. Each Delphi round will be developed and piloted. This approach has been utilized in the past study [19].To ensure consistency and standardization among all stakeholder evaluators, several measures will be implemented. All participants will receive orientation materials, including guidance on the 9-point likert scale and outcome definitions in lay language, with medical terms included in brackets, to ensure comprehension across diverse stakeholder groups. A brief training session (either virtual or in-person) will be conducted, and a facilitator will guide the consensus meeting to ensure uniform application of evaluation criteria prior to rescoring.

**1) OSF patients:** OSF patients will be chosen at random from the aforementioned centers, and they will not be the same who were participated in the focus groups and qualitative interviews.
**2) Clinician sample:**The membership lists of specialists from the Indian Academy of Oral Medicine & Radiology and the Indian Dental Association,specialists, clinical researchers will be used to compile the clinician sample. We will email the clinicians and request their participation in the surveys, but only if they agree to take part in all three rounds.
**3) Researchers/experts sample:** Researchers will be identified through relevant publications and purposively selected based on their wide range of expertise in the fields of OSF and patient-centered research. Selection criteria will include not only by scholarly contributions but also by clinical experience, involvement in patient care, multidisciplinary engagement, and professional recognition in the field.

### Delphi surveys

#### Round 1.

Participants will be asked to rate the significance of each result using the GRADE Working Group’s suggested 9-point Likert scale, with scores of 1–3 denoting “not important,” 4–6 denoting “important but not critical,” and 7–9 denoting “critical” [20]. There will be free-text sections where participants can add any other outcomes, they feel are crucial. Then each item will receive descriptive statistics. The study team will discuss any new findings gleaned from the free-text fields. Round 2 will include all results from round 1.

#### Round 2.

Participants will be contacted, asked to complete the Delphi questionnaire once more, and informed of their round one results. Additionally, they will see the mean scores supplied for each outcome by their stakeholder group alone. For every result, descriptive statistics will be computed. For round three, all results will be carried over.

#### Round 3.

The results will be scored again by the participants. Participants will see their findings as well as the average scores for each stakeholder group in this last round.

Every participant will receive a reminder two weeks after the initial request to do an online activity for each level of the Delphi within four weeks. Response rates will be tracked, and participants will be encouraged to respond by receiving follow-up emails and phone calls.

#### Final data analysis.

Each stakeholder group’s percentage of the total scores will be determined as follows: 1–3, 4–6, and 7–9. Every outcome score will be considered “consensus in” if more than 70% of participants gave it a score of 7–9 and less than 30% gave it a score of 1–3. “Consensus out” occurs when more than 70% of participants give a result a score of 1–3, and less than 15% of participants give it a score of 7–9. According to earlier studies, any other combinations will be regarded as “no consensus” [17,19].

### Step4- Consensus meeting

Finally, consensus discussions will be convened with patients, clinicians, and researchers/experts as key participants. To increase the acceptability and execution of the COS, we will make an effort to ensure that each stakeholder group is adequately represented. The Delphi procedure, which was selected as the most effective and practical way to include the important stakeholders from different settings, backgrounds, and geographic locations, would be used to try and reach consensus. Purposive sampling will be used to choose the important stakeholders who completed the Delphi survey and are available to attend the meeting. Every attempt will be made to draw a diverse sample of participants, with about equal numbers of participants drawn from each stakeholder group. The meeting will be facilitated by a researcher. Each outcome’s results will be given in turn during the consensus meeting, followed by discussion and rescoring utilizing an electronic scoring system that is completely anonymous. The outcomes of the consensus meeting will be evaluated against the established consensus definition in order to develop the final COS.

### Dissemination

Peer-reviewed journal publications, public health meetings, and national and international scientific events will all be used to communicate the results of this study. In the COMET database, the finished COS will be made available. The research team will create a user-friendly summary of the research findings that will be distributed to all the study’s stakeholders.

### Status and timeline of the study

After finishing the systematic review, we are now preparing to initiate patient recruitment forfocus groups and in-depth interviews.Patient data collection is expected to be completed in one month, followed by the Delphi survey, which will conclude in approximately 1.5 months. The COS will then be finalized within 15 days at the end of the study.

## Discussion

The multi-step procedure that will be utilized to develop a COS for OSF management is described in this protocol paper. Future research in this field is expected to benefit from the development of a standardized set of outcomes in terms of study design, execution, and reporting. The bias in outcome reporting will be lessened because only the agreed outcomes will be collected and reported in subsequent research.Developing this COS will also aid with reducing heterogeneity among upcoming research, which can improve their comparability and, eventually, make it easier to synthesize the data in systematic reviews and meta-analyses. Then, it will be possible to draw firm results and deliberate on the oral health measures that are most successful for this cohort. Additionally, the application of the intended COS will be ensured by involving important stakeholders at every stage of the process. Numerous stakeholders, such as patients, healthcare workers, and decision-makers, will find the outcomes to be pertinent and significant.

### Clinical implications

The creation of OSFCOS will enhance the reporting of outcomes in OSF trials which will increase their research quality and comparability. This will enhance the synthesis of evidence, support clinical decisions, and assist in the crafting of comprehensive treatment guidelines. The participation of patients and professionals validates the relevance and applicability of the COS. This initiative has the potential to diminish unnecessary research activity, enhance the care provided to patients, and serve as an exemplar for COS development concerning other oral health issues.

## Supporting information

S1 Table Core Outcome Set-STAndardised Protocol items: The COS- STAP statement. (DOCX)
