# Supplementary material for: Development of a core outcome set for trials for management of oral submucous fibrosis (OSFCOS): A consensus study protocol
Source: PLoS One. 2025 Jul 30;20(7):e0325158. doi: 10.1371/journal.pone.0325158 (PMC12309984; doi:10.1371/journal.pone.0325158)
Supplement: S1 Table — (DOCX) [file pone.0325158.s001.docx]

| **TITLE/ABSTRACT** |  |  | **Page** |
| --- | --- | --- | --- |
| **Title** | 1a | Identify in the title that the paper describes the protocol for the planned development of a COS | 1 |
| **Abstract** | 1b | Provide a structured abstract | 2 |
| **INTRODUCTION** |  |  |  |
| **Background and**  **objectives** | 2a | Describe the background and explain the rationale for developing the COS, and identify the reasons why a COS is needed and the potential barriers to its implementation | 3-4 |
|  | 2b | Describe the specific objectives with reference to developing a COS | 5 |
| **Scope** | 3a | Describe the health condition(s) and population(s) that will be covered by the COS | 4-5 |
|  | 3b | Describe the intervention(s) that will be covered by the COS | 4-5 |
|  | 3c | Describe the context of use for which the COS is to be applied | 5 |
| **METHODS** |  |  |  |
| **Stakeholders** | 4 | Describe the stakeholder groups to be involved in the COS development process, the nature of and rationale for their involvement and also how the individuals will be identified; this should cover involvement both as members of the research team and as participants in the study | 6-10 |
| **Information sources** | 5a | Describe the information sources that will be used to identify the list of outcomes. Outline the methods or reference other protocols/papers | 5-6 |
|  | 5b | Describe how outcomes may be dropped/combined, with reasons | 6-11 |
| **Consensus**  **process** | 6 | Describe the plans for how the consensus process will be undertaken | 8-11 |
| **Consensus**  **definition** | 7a | Describe the consensus definition | 8-11 |
| **ANALYSIS** | 7b | Describe the procedure for determining how outcomes will be added/combined/dropped from consideration during the consensus process | 8-11 |
| **Outcome**  **scoring/feedback** | 8 | Describe how outcomes will be scored and summarised, describe how participants will receive feedback during the consensus process | 8-11 |
| **Missing data** | 9 | Describe how missing data will be handled during the consensus process | 9-10 |
| **ETHICS and DISSEMINATION** |  |  |  |
| **Ethics**  **approval/informed consent** | 10 | Describe any plans for obtaining research ethics committee/institutional review board approval in relation to the consensus process and describe how informed consent will be obtained (if relevant) | 5 |
| **Dissemination** | 11 | Describe any plans to communicate the results to study participants and COS users, inclusive of methods and timing of dissemination | 11 |
| **ADMINISTRATIVE INFORMATION** |  |  |  |
| **Funders** | 12 | Describe sources of funding, role of funders | 12 |
| **Conflicts of**  **interest** | 13 | Describe any potential conflicts of interest within the study team and how they will be managed | 12 |
